# Analysis of apoB Concentrations Across Early Adulthood and Predictors for Rates of Change Using CARDIA Study Data

**DOI:** 10.1016/j.jlr.2022.100299

**Published:** 2022-10-19

**Authors:** John T. Wilkins, Hongyan Ning, Allan Sniderman, Neil Stone, James Otvos, David R. Jacobs, Ravi Shah, Venkatesh L. Murthy, Jamal Rana, Norrina Allen, Donald M. Lloyd-Jones

**Affiliations:** 1 Department of Medicine (Cardiology), Northwestern University Feinberg School of Medicine, Chicago, Illinois, USA; 2 Department of Preventive Medicine, Northwestern University Feinberg School of Medicine, Chicago, Illinois, USA; 3 Mike and Valeria Rosenbloom Centre for Cardiovascular Prevention, Department of Medicine, McGill University Health Centre, Montreal, Quebec, Canada; 4 NMR Diagnostics, Laboratory Corporation of America Holdings (LabCorp), Morrisville, North Carolina, USA; 5 Division of Epidemiology and Community Health, School of Public Health, University of Minnesota, Minneapolis, USA; 6 Department of Medicine, Division of Cardiology, Vanderbilt University School of Medicine Nashville, Tennessee, USA; 7 Department of Medicine and Radiology, University of Michigan, Ann Arbor, Michigan, USA; 8 Department of Cardiology, Kaiser Permanente Oakland Medical Center, California, USA

**Keywords:** apolipoprotein B, epidemiology, change, young adults, apoB, apolipoprotein B100, ASCVD, atherosclerotic cardiovascular disease, CARDIA, Coronary Artery Risk Development in Young Adults, DM, diabetes mellitus, HEI, Healthy Eating Index, LDL-P, LDL particle, Lp(a), lipoprotein (a), NHANES, National Health and Nutrition Examination Survey, PA, physical activity, TG, triglyceride, Y0, year 0

## Abstract

The cumulative exposure to apolipoprotein B (apoB)-containing lipoproteins in the blood during early adult life is a central determinant of atherosclerotic cardiovascular disease risk. To date, the patterns and rates of change in apoB through early adult life have not been described. Here, we used NMR to measure apoB concentrations in up to 3055 Coronary Artery Risk Development in Young Adults (CARDIA) Study participants who attended the years 2 (Y2), 7 (Y7), 15 (Y15), 20 (Y20), and 30 (Y30) exams. We examined individual-level spaghetti plots of apoB change, and we calculated average annualized rate of apoB concentration change during follow-up. We used multivariable linear regression models to assess the associations between CARDIA participant characteristics and annualized rates of apoB change. Male sex, higher measures of adiposity, lower HDL-C, lower Healthy Eating Index, and higher blood pressures were observed more commonly in individuals with higher apoB level at Y2 and Y20. Inter- and intra-individual variation in apoB concentration over time was substantial—while the mean (SD) rate of change was 0.52 (1.0) mg/dl/year, the range of annualized rates of change was −6.26 to +9.21 mg/dl/year. At baseline, lower first apoB measurement, female sex, White race, lower BMI, and current tobacco use were associated with apoB increase. We conclude that the significant variance in apoB level over time and the modest association between baseline measures and rates of apoB change suggest that the ability to predict an individual’s future apoB serum concentrations, and thus their cumulative apoB exposure, after a one-time assessment in young adulthood is low.

Apolipoprotein B100 (apoB) is the primary structural protein and binding ligand for the atherogenic lipoprotein particles: VLDL, remnant, IDL, LDL, and lipoprotein (a) (Lp(a)) (1, 2). Each atherogenic lipoprotein particle contains one molecule of apoB. Genetic polymorphisms at different loci that mediate lipid metabolism (i.e., *LDLR, LPA*, and others) and differences in LDL-receptor density, cholesterol and triglyceride (TG) exchange, insulin resistance, and lifestyle factors result in substantial interindividual differences in the number of apoB-containing particles for a given total mass of cholesterol and TG (3, 4). Thus, measurement of apoB concentration captures the aggregate burden of atherogenic lipoprotein particles present in the blood more precisely than the measurement of the cholesterol concentration in LDL (LDL-C) or non-HDL (non-HDL-C).

The retention of apoB-containing lipoproteins in the subendothelial space, as well as the chronic inflammatory response to these particles, is central to the pathogenesis of atherosclerosis and subsequent atherosclerotic cardiovascular disease (ASCVD) events (5, 6). ApoB blood concentrations have strong associations with subclinical atherosclerosis and incident ASCVD (7). Because of its ability to quantify the burden of atherogenic lipoprotein particle number more completely and accurately than non-HDL-C, multiple studies suggest that apoB is a superior marker of ASCVD risk, particularly in the 8–20% of people who have higher or lower than average apoB levels for a given non-HDL-C level (8). Nonetheless, other clinical markers of atherogenic lipoprotein burden like non-HDL-C and LDL-C are commonly used in clinical practice.

Since apoB is a causal determinant of atherosclerosis (9), and exposure to atherogenic lipoproteins across early adult life is a strong predictor of ASCVD risk (10, 11), it is important to understand normative apoB concentrations in young adults, their patterns and rates of change through midlife, and the predictors of change. Furthermore, understanding how these rates of change compare to other commonly used measures of atherogenic burden may help inform the clinical utility of apoB measurement in young adults, as more stable measures may serve as more reliable markers of an individual patient’s expected future burden of atherogenic lipoproteins, and they may perform better as long-term ASCVD risk markers in young adults. To date, the patterns and rates of change in apoB concentrations within the same individuals across young adulthood have not been described. Here, we report the first descriptions of the rates of intraindividual apoB change, as well as predictors of change, and we compare these rates of change for other commonly used measures of atherogenic lipoprotein burden, across early adult life using unique data from over 3,055 Coronary Artery Risk Development in Young Adults (CARDIA) study participants.

## Materials and Methods

### Study sample

The CARDIA study recruited 5,115 black and white men and women aged 18–30 years in 1985–1986 from four sites across the United States: Birmingham, AL; Chicago, IL; Minneapolis, MN; and Oakland, CA. Participants were sampled to achieve a cohort balanced by race (52% black and 48% white), sex (55% female and 45% male), education (40% with 12 years and/or younger, 60% with more than 12 years of education), and age (45% 18–24 years and 55% 25–30 years). CARDIA participants have undergone in-person examinations at baseline (year 0: Y0) and at Y2, Y5, Y7, Y10, Y15, Y20, Y25, and Y30 (12). Retention rates among surviving participants at each in-person examination have been high, at 91, 86, 81, 79, 74, 72, 72, and 71%, respectively. Contact is maintained with participants via telephone, mail, or e-mail every 6 months, with annual medical history ascertainment between in-person examinations. Over the last 5 years, >90% of the surviving cohort members have been directly contacted, and follow up for vital status is virtually complete through related contacts and intermittent National Death Index searches.

### Inclusion criteria

Since we were interested in understanding the natural history of apoB concentration change across early adulthood, we included samples from participants who had samples available at Y20 and at least two of three from Y15, Y7, or Y2 (*n* = 3,055). For patients who met these inclusion criteria, we also included data from Y30 when it was available (*N* = 2,474). Of the 3,055, we measured apoB in all those with available serum or plasma samples at Y2 (*N* = 1,881), Y7 (*N* = 2,582), Y15 (*N* = 2,474), Y20 (*N* = 3,055), and Y30 (*N* = 2,474). The characteristics of included and excluded participants are presented in supplemental Table S2.

### Traditional risk factor and lifestyle measurement

Age, race, and sex were determined via self-report. Height, weight, and waist circumference were measured with the participants in light clothing using a standardized stadiometer, tape measure, and calibrated scale; BMI (kg/m^2^) was calculated. Detailed dietary data are available for Y0, Y7, and Y20. Dietary data obtained at baseline were used to represent Y2 dietary patterns. The CARDIA dietary data were obtained via an interview-administered method that included a short questionnaire regarding general dietary practices followed by a comprehensive questionnaire about typical intake of foods (about 100 header questions such as “do you eat meat,” followed by open-ended responses in answer to that header question). Detailed data on portion sizes, frequency of consumption, and common additives were conducted on foods that were regularly consumed as well (12, 13). Dietary data were designed to reflect the habitual intake (past month) of CARDIA participants. From these diet history data, the Healthy Eating Index (HEI) at Y2 and Y20 was calculated and used to represent overall dietary quality (14).

At each CARDIA examination, participants were given the interviewer-administered Physical Activity (PA) History Questionnaire. In brief, participants were asked about self-reported leisure time activity and the frequency of participation in 13 specific PA categories (eight of vigorous and five of moderate intensity) of recreational sports, structured exercise, home maintenance, and occupational activities during the preceding 12 months. Intensity of each activity was expressed as metabolic equivalents (15, 16). The PA score summed frequency time intensity over the 13 activities to get total activity (in exercise units).

Blood pressure was measured after 5 min of rest in the seated position using a random zero mercury sphygmomanometer, replaced from Y20 forward with an Omron oscillometer (calibrated to the random zero). The means of the second and third systolic and diastolic measurements were used. Alcohol intake, smoking habits, and educational attainment were determined with the use of standardized and validated questionnaires. Blood pressure- and cholesterol-lowering medication use was determined by self-report.

After a 12-h fast, blood was drawn from a vein in the antecubital fossa into a Vacutainer, coated with EDTA for plasma. Serum and plasma samples were obtained and stored at −80°C for future analysis. Plasma samples were transported on dry ice to the Northwest Lipid Research Center in Seattle, Washington. Plasma concentrations of total cholesterol and TGs were measured using a standard enzymatic assay. HDL-C was quantified after precipitation with dextral sulfate-magnesium chloride on ABA 200 Biochromatic instrument (Abbott Laboratories, North Chicago, IL). LDL-C was calculated using the Friedewald equation (17) and non-HDL-C by the difference between total cholesterol and HDL-C.

ApoB and LDL particle (LDL-P) were measured from frozen serum (Y2) and EDTA plasma (Y7, Y15, Y20, and Y30) samples by NMR spectroscopy at Labcorp (Morrisville, NC) using the high-throughput Vantera® Clinical NMR Analyzer platform. ApoB was quantified using partial least squares regression modeling of the lipid methyl and methylene spectral region as previously described (18). ApoB concentrations produced by this assay have been extensively validated as equivalent to those measured by immunoassay (*R* = 0.98), with precision and accuracy verified quarterly by blinded Centers for Disease Control and Prevention Lipids Standardization Program proficiency testing (18). LDL-P was quantified by *NMR LipoProfile*® analysis using the LP4 deconvolution algorithm (18, 19).

Only serum was available for the Y2 samples. Because of well-described dilution effects that occur in EDTA plasma tubes (20), we applied a previously derived CARDIA-specific correction factor for total cholesterol of 0.9666 to the NMR-derived apoB and LDL-P measures that were obtained from the Y2 serum separator tubes.

### Statistical analysis

To describe the characteristics associated with different apoB concentrations in midlife, we stratified participants into quartiles of apoB concentration at Y20 and showed the participant characteristics by Y20 quartiles at the Y2, Y7, Y15, Y20, and Y30 exams. We compared demographics, anthropometrics, lifestyle behaviors, and traditional risk factor data across quartiles using ANOVA and Chi-square tests as appropriate.

To understand the shifts in apoB concentration distribution over time in CARDIA, we generated separate distributions of apoB at exam years 2, 7, 15, 20, and 30. To visualize individual-level change in apoB, LDL-P, LDL-C, and non-HDL-C concentrations, we generated spaghetti plots of the individual participant’s lipoprotein concentrations across exams. Since the overall pattern of change appeared mostly linear, we calculated annualized rates of lipoprotein change. Individual annualized rates of change were calculated by subtracting the last measured atherogenic lipoprotein measure (Y20 or Y30) value by the first measured value. This difference in atherogenic lipoprotein level was then divided by the time between the first and last atherogenic lipoprotein measurement value. We then calculated separate quartiles of atherogenic lipoprotein annualized rate of change, and Y2, Y20, and Y30 characteristics were compared across quartiles. We also created a distribution of atherogenic lipoprotein change as well as individual-level waterfall plot of annualized rate of change. To compare intraindividual variability over time, we generated distributions of the percent annual change in each lipid measure.

We used linear regression models to assess the associations between characteristics and rates of apoB change. Models were adjusted as follows: model 1: first measurement of apoB to account for the level at which the participant’s apoB began; model 2: model 1 + demographics; model 3: model 2 + commonly measured clinical characteristics at baseline (HDL-C, systolic blood pressure, BMI, blood pressure-lowering therapy, diabetes mellitus [DM] status, current tobacco use, PA [exercise units], serum glucose, and diet [HEI]); and model 4: model 2 + yearly averages (for characteristics not assessed at each exam) or cumulative values (for those characteristics assessed at all exams) of the commonly measured clinical characteristics used in model 3. Multicollinearity was tested between the selected independent variables using the variance inflation factor, and none was found.

To assess the association of lipid-lowering therapy during follow-up, we repeated the analyses outlined above after removing all participants (*N* = 717) who reported taking lipid-lowering pharmacotherapy from examination Y2 though Y30 as a sensitivity analysis. Furthermore, more than 2,000 participants did not meet NMR analysis criteria and were not included in the primary analysis. To impute the missing factors, we conducted multiple imputation by chained equations with fully conditional specification using the SAS MI package (SAS Institute, Cary, NC) (21). NMR lipid measurements and related risk factors from all visits were included sequentially in the model specification. We excluded those participants who died prior to exam year 7 (two exam visits for annualized apoB change calculation), and finally, 5,066 participants were included in the imputation model. We created 10 imputed datasets, and the imputed values were set to missing after participants died. The distribution of covariates between the observed and imputed dataset was similar. Each regression analysis described above was performed separately in each of the imputed datasets, and the results were combined using Rubin’s rules. SAS, version 9.4, was used for the analysis (22).

## Results

### Cohort participant characteristics

Participant characteristics at Y2 and Y20, stratified by quartile of apoB concentration at Y20, are presented in Tables 1 and 2, respectively. The mean (SD) apoB at the Y2 exam was 85.2 (20.2) mg/dl. At the Y2 exam, participants in the higher Y20 apoB quartiles were more likely to be men, have a higher BMI, greater waist circumference, higher blood pressure, and have lower HEI. Those in the higher Y20 apoB strata had lower HDL-C and higher Y2 apoB, LDL-C, non-HDL-C, and TG levels as well.

**Table 1:** Exam year 2—characteristics of CARDIA participants by exam year 20 apoB concentration quartile

Variable	ApoB concentration quartile (*N*) (range)				*P*
	0–25% (*n* = 763) (33.1–81.7 mg/dl)	25–50% (*n* = 764) (81.7–93.9 mg/dl)	50–75% (*n* = 764) (93.9–108.0 mg/dl)	75–100% (*n* = 764) (108.0–196.8 mg/dl)	
Black, %	354 (46.4%)	354 (46.3%)	341 (44.6%)	322 (42.1%)	0.30
Male, %	298 (39.1%)	297 (38.9%)	339 (44.4%)	407 (53.3%)	<0.01
Age, years	27.0 (3.6)	27.1 (3.6)	27.3 (3.5)	27.3 (3.5)	0.21
Educational status, years	14.5 (2.4)	14.5 (2.3)	14.5 (2.3)	14.3 (2.4)	0.38
ApoB concentration, mg/dl^a^	72.9 (17.7)	82.8 (18.0)	86.9 (16.6)	97.1 (20.3)	<0.01
Total cholesterol, mg/dl	161.0 (30.9)	176.6 (31.2)	181.7 (30.1)	199.5 (34.5)	<0.01
HDL-C, mg/dl	50.9 (13.1)	50.7 (12.2)	48.9 (11.9)	48.0 (12.8)	<0.01
Non-HDL-C, mg/dl	110.1 (30.3)	125.9 (29.6)	132.8 (27.8)	151.5 (33.5)	<0.01
LDL-C, mg/dl	95.9 (28.2)	111.9 (28.5)	118.2 (26.6)	134.7 (30.9)	<0.01
LDL-P concentration, nmol/l	962.3 (317.9)	1120.2 (304.6)	1191.2 (299.7)	1368.8 (358.8)	<0.01
TG concentration, mg/dl	77.6 (44.3)	78.6 (44.5)	82.5 (42.4)	96.6 (59.5)	<0.01
BMI, kg/m^2^	24.6 (5.3)	25.1 (5.4)	25.2 (4.8)	25.5 (5.0)	<0.01
Waist circumference, cm	77.8 (12.3)	79.0 (12.5)	80.0 (11.2)	81.7 (11.4)	<0.01
Systolic blood pressure, mm Hg	106.6 (10.8)	107.0 (10.5)	107.3 (10.1)	109.4 (10.7)	<0.01
Diastolic blood pressure, mm Hg	66.8 (9.1)	67.0 (9.7)	67.4 (9.2)	68.7 (9.0)	<0.01
Serum glucose, mg/dl	85.4 (18.4)	84.5 (13.4)	85.0 (10.6)	86.0 (14.0)	0.38
PA intensity score	397.3 (296.0)	369.7 (284.3)	377.1 (277.2)	394.7 (287.4)	0.18
HEI score (0–100)	62.9 (9.7)	62.4 (9.4)	62.3 (9.1)	61.3 (9.3)	<0.01
Saturated fats (% of energy)	14.0 (3.1)	14.1 (2.9)	14.2 (2.8)	14.2 (2.9)	0.57
Proteins (% of energy)	14.9 (2.5)	14.8 (2.7)	14.9 (2.6)	14.9 (2.7)	0.97
Carbohydrates (% of energy)	46.4 (7.7)	46.3 (7.4)	45.4 (6.7)	45.8 (7.3)	0.03
Hypertension treatment, %	13 (1.7%)	28 (3.7%)	21 (2.8%)	16 (2.2%)	0.08
Diabetes treatment, %	9 (1.2%)	7 (0.9%)	2 (0.3%)	11 (1.5%)	0.10
Prevalent diabetes, %	11 (1.5%)	11 (1.5%)	6 (0.8%)	16 (2.1%)	0.21
Prevalent hypertension, %	18 (2.4%)	39 (5.2%)	30 (4.0%)	25 (3.3%)	0.04
Current smoking, %	183 (24.7%)	173 (23.1%)	192 (25.9%)	209 (28.0%)	0.16
Regular alcohol use, %	462 (71.4%)	446 (70.6%)	455 (70.7%)	460 (73.7%)	0.57

Continuous measures presented as mean and SD in parentheses. Categorical variables are presented as number and percentage in parentheses.

a Mean apoB concentration derived from the 1,881 participant samples that were analyzed via NMR.

**Table 2:** The Y20 characteristics of CARDIA participants by the Y20 examination apoB concentration quartile

Variable	ApoB concentration quartile (*N*) (range)				*P*
	0–25% (*n* = 763) (33.1–81.7 mg/dl)	25–50% (*n* = 764) (81.7–93.9 mg/dl)	50–75% (*n* = 764) (93.9–108.0 mg/dl)	75–100% (*n* = 764) (108.0–196.8 mg/dl)	
Black, %	354 (46.4%)	354 (46.3%)	341 (44.6%)	322 (42.1%)	0.30
Male, %	298 (39.1%)	297 (38.9%)	339 (44.4%)	407 (53.3%)	<0.01
Age, years	45.0 (3.6)	45.2 (3.6)	45.3 (3.6)	45.5 (3.5)	0.07
Educational status, years	15.2 (2.6)	15.1 (2.5)	15.1 (2.5)	14.8 (2.5)	<0.01
ApoB, mg/dl	71.9 (8.2)	88.0 (3.5)	100.4 (4.1)	123.4 (13.4)	<0.01
Total cholesterol, mg/dl	153.9 (20.7)	177.9 (17.9)	195.5 (17.0)	228.8 (25.2)	<0.01
HDL-C, mg/dl	56.2 (15.3)	55.9 (15.4)	53.3 (15.1)	49.7 (14.0)	<0.01
Non-HDL-C, mg/dl	97.7 (16.4)	122.0 (13.0)	142.2 (12.3)	179.1 (23.9)	<0.01
LDL-C, mg/dl	81.4 (14.8)	104.8 (12.1)	123.0 (12.0)	152.2 (24.0)	<0.01
LDL-P concentration, nmol/l	891.0 (175.6)	1157.2 (138.1)	1384.1 (148.9)	1780.3 (279.2)	<0.01
TG concentration, mg/dl	86.6 (52.7)	95.5 (54.1)	107.8 (59.3)	151.5 (104.9)	<0.01
BMI, kg/m^2^	27.9 (7.3)	29.0 (7.0)	29.7 (6.5)	30.7 (6.7)	<0.01
Waist circumference, cm	87.5 (16.5)	90.5 (15.6)	92.7 (14.2)	96.5 (14.5)	<0.01
Systolic blood pressure, mm Hg	113.8 (14.9)	114.9 (15.3)	115.3 (13.8)	118.6 (13.8)	<0.01
Diastolic blood pressure, mm Hg	70.2 (11.6)	71.5 (11.2)	72.4 (10.6)	74.6 (10.4)	<0.01
Serum glucose, mg/dl	90.1 (21.4)	91.2 (23.9)	90.1 (16.1)	95.5 (29.3)	<0.01
PA intensity score	352.7 (279.3)	344.7 (288.9)	319.7 (254.4)	335.1 (279.0)	0.11
HEI score (0–100)	70.8 (10.9)	70.1 (10.2)	69.7 (10.2)	68.6 (10.1)	<0.01
Saturated fats (% of energy)	11.3 (3.2)	11.5 (3.2)	11.6 (3.4)	11.7 (2.9)	0.12
Protein (% of energy)	15.5 (3.6)	15.5 (3.8)	15.5 (3.6)	15.7 (3.7)	0.60
Carbohydrates (% of energy)	47.3 (9.4)	46.8 (9.7)	46.7 (9.4)	47.0 (9.7)	0.65
Hypertension treatment, %	121 (15.9%)	126 (16.5%)	132 (17.3%)	153 (20.0%)	0.15
Diabetes treatment, %	69 (9.0%)	59 (7.7%)	47 (6.2%)	56 (7.3%)	0.20
Prevalent diabetes, %	74 (9.7%)	64 (8.4%)	54 (7.1%)	73 (9.6%)	0.22
Prevalent hypertension, %	151 (19.8%)	166 (21.8%)	164 (21.5%)	189 (24.7%)	0.13
Current smoking, %	132 (17.5%)	120 (15.8%)	157 (20.7%)	158 (20.9%)	0.03
Regular alcohol use, %	419 (56.1%)	399 (53.1%)	408 (54.7%)	403 (53.9%)	0.69

Continuous measures presented as mean and SD in parentheses. Categorical variables are presented as number and percentage in parentheses.

Participant characteristics at Y20, stratified by quartile of apoB concentration at Y20, are presented in Table 2. The mean (SD) apoB level was 95.9 (20.5) mg/dl at the year 20 exam. As was seen for the Y2 characteristics, when compared with those in lower quartiles of apoB level, CARDIA participants with higher apoB levels at Y20 exam (mean age = 45 years) were more likely to be men and have higher BMI, waist circumference, and blood pressures. Participants with higher apoB at Y20 were also more likely to have lower HDL-C and higher glucose. As expected, the cholesterol and TG concentrations in the apoB lipoproteins were higher as well. There were no significant differences in self-reported saturated fat intake, though the HEI was lower in higher apoB groups. There were no significant differences in self-reported PA level at Y20 across apoB quartiles.

The characteristics for exam Y7, Y15, and Y30 are presented in supplemental Table S1A–C. The associations between characteristics and apoB quartile were similar in Y7, Y15, and Y30 when compared with Y20, though the absolute differences in these characteristics at Y7 and Y15 were smaller than were observed at Y20 and Y30.

### Distributions of apoB

**Fig. 1:**
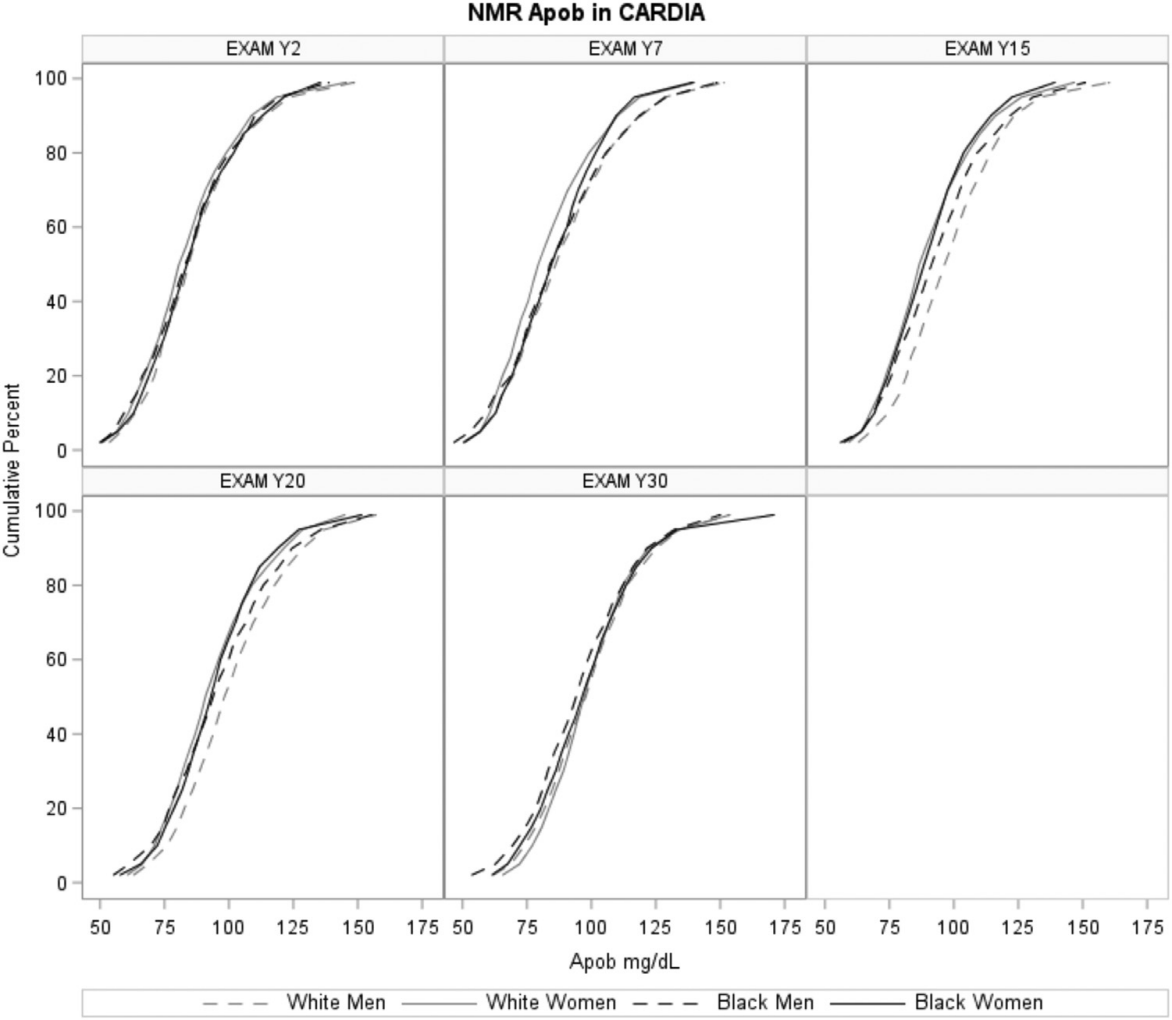
The distribution of apoB concentration by examination year and sex/race groups in CARDIA.

The distributions of apoB stratified by race and sex across exam years 2, 7, 15, 20, and 30 are presented in Fig. 1. The mean (SD) apoB concentrations at exam years 2, 7, 15, 20, and 30 were 85.2 (20.2), 85.8 (21.5), 92.7 (20.2), 95.9 (20.5), and 97.9 (20.3) mg/dl, respectively.

### Correlations between apoB and other measures of atherogenic lipoproteins

As expected, the correlation coefficients for apoB and non-HDL-c, LDL-C, and LDL-P measured at the Y20 exam are 0.89, 0.87, and 0.94, respectively. The correlations are similar across exam years 2, 7, 15, and 30.

### Intraindividual change in apoB over time

**Fig. 2:**
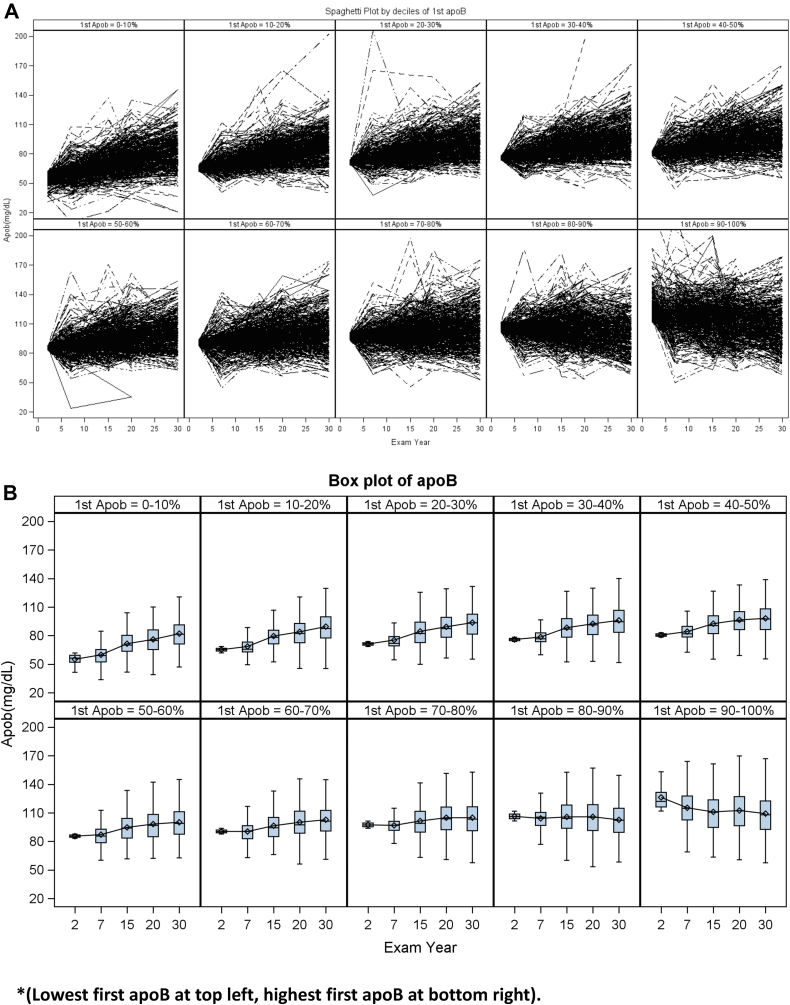
Change in apoB from first apoB measurement (exam 2 or 7) through the Y30 exam stratified by decile of first apoB measurement presented as spaghetti plots (panel A) and box plots (panel B). ∗(Lowest first apoB at top left and highest first apoB at bottom right).

Spaghetti plots representing individual-level change over time for CARDIA participants, stratified by the decile of their first apoB concentration measurement, are presented in Fig. 2.

Qualitatively, across all strata of first baseline apoB measurement, the interindividual variance in apoB appears to increase over the early adult life course (i.e., there is a widening distribution over time among individuals with very similar baseline apoB concentrations). The pattern of change for most participants appears to be fairly linear, with those in the lower baseline level more likely to increase over time and those in highest strata somewhat more likely to decrease over time. Although several outlier measurements are present for each exam year, on subsequent measurements, the values for those individuals appear more normative. Patterns of change were similar for non-HDL-C, LDL-C, and LDL-P though the absolute ranges of these values at baseline and at Y30 were greater (data not shown).

### Distribution of annualized rate of change

**Fig. 3:**
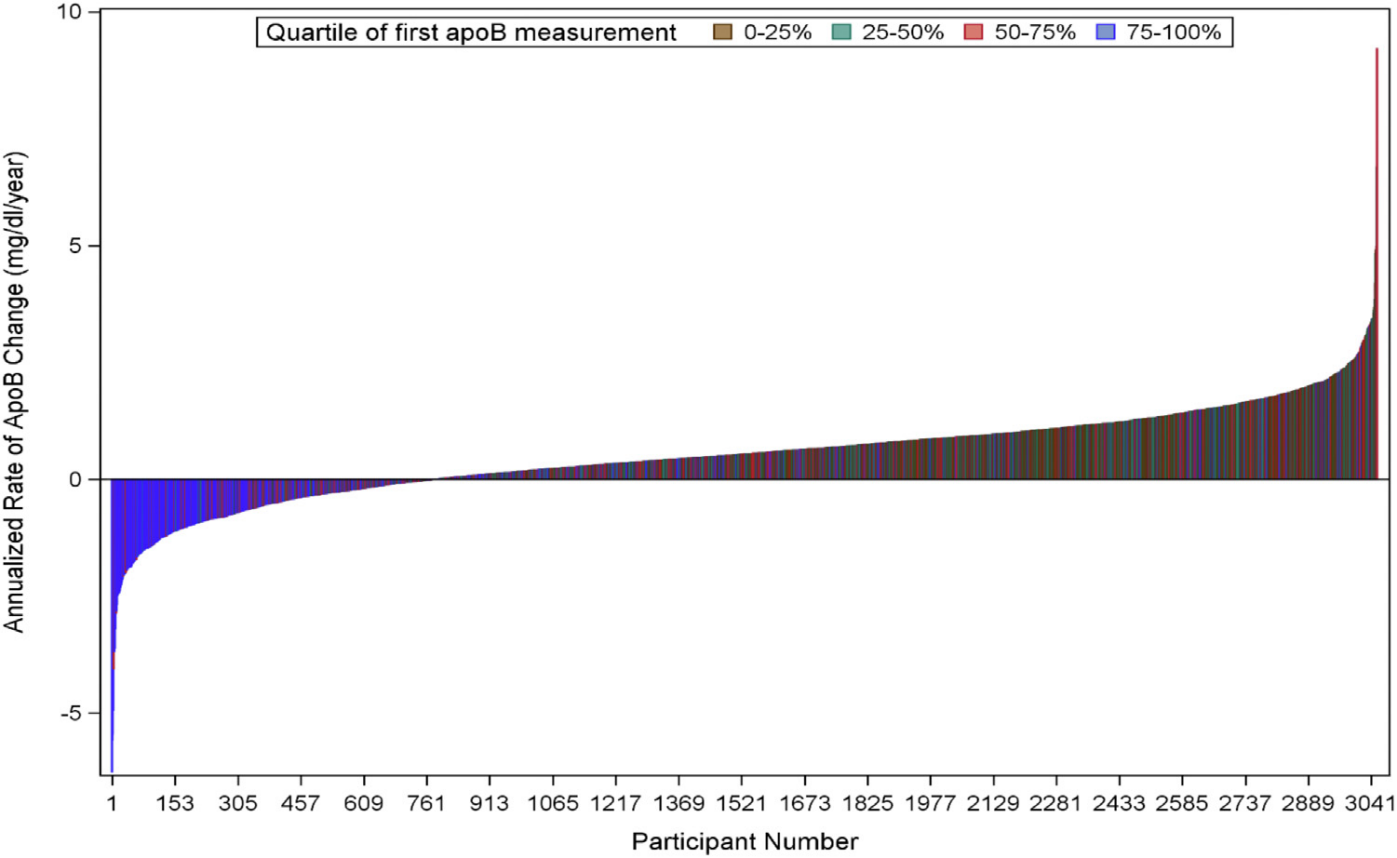
Waterfall plot of the individual-level annualized rate of apoB change.

The waterfall plot of individual-level annualized rate of absolute change in apoB is presented in Fig. 3. The mean (SD) rate of change was 0.52 (1.00) mg/dl/year, with a range of −6.2 to 9.2 mg/dl/year from a participant mean age of 27 through 45 years. White females had a slightly higher average mean rate of change, at +0.64 mg/dl/year (95% CI: 0.57–0.71), than observed in all other race/sex groups (mean rate and 95% CI: black male = +0.40 [+0.32 to +0.49], white male = +0.47 [+0.40 to +0.54], and black female = +0.50 [+0.43 to +0.56] mg/dl/year). The distributions of annualized rates of change in sex and race groups are presented in supplemental Fig. S1.

The Y2 characteristics of quartiles of annualized rate of change quartiles are presented in Table 3. The mean (SD) rates of change in the lowest to the highest quartile of change were −0.7 (0.7), +0.3 (0.2), +0.8 (0.2), and +1.7 (0.6) mg/dl/year, respectively. Notably, the quartiles with the lowest rate of increase (−0.7 mg/dl/year) had the highest initial apoB concentration of 102.7 (21.0) mg/dl, and the group with the highest rate of increase (+1.7 mg/dl/year) had the lowest starting apoB concentration of 76.1 (16.1) mg/dl. Participants in the higher rate of increase quartiles were more likely to be women and have lower BMI, waist circumference, glucose, blood pressure, higher PA and HEI, and a lower prevalence of DM than was observed in the lowest rate of change quartile (Table 3).

**Table 3:** Y2 characteristic comparison by quartile of annualized apoB concentration change (last–first apoB/years between measurements)

Variable	0–25% (*n* = 763)	25–50% (*n* = 764)	50–75% (*n* = 764)	75–100% (*n* = 764)	*P*
Annualized apoB change, mg/dl/year	−0.7 (0.7)	0.3 (0.2)	0.8 (0.2)	1.7 (0.6)	<0.01
First apoB, mg/dl	102.7 (21.0)	84.8 (16.9)	77.9 (15.6)	76.1 (16.1)	<0.01
Last apoB, mg/dl	86.1 (17.0)	91.7 (16.9)	98.1 (15.7)	114.6 (20.8)	<0.01
Black, %	361 (47.3%)	363 (47.5%)	327 (42.8%)	320 (41.9%)	0.05
Male, %	364 (47.7%)	361 (47.3%)	310 (40.6%)	306 (40.1%)	<0.01
Age, years	27.6 (3.5)	27.0 (3.6)	27.0 (3.6)	27.0 (3.6)	<0.01
Educational status, years	14.3 (2.4)	14.4 (2.3)	14.7 (2.3)	14.3 (2.3)	0.02
Total cholesterol, mg/dl	204.7 (38.2)	177.7 (29.5)	169.3 (28.1)	167.8 (28.0)	<0.01
HDL-C, mg/dl	49.2 (13.5)	49.8 (12.7)	50.0 (11.2)	49.3 (12.7)	0.68
Non-HDL-C, mg/dl	155.5 (36.8)	127.9 (29.1)	119.3 (27.0)	118.5 (27.4)	<0.01
LDL-C, mg/dl	137.6 (34.6)	113.5 (28.0)	105.7 (25.8)	104.8 (26.4)	<0.01
LDL-P concentration, nmol/l	1423.0 (392.6)	1143.5 (310.5)	1050.3 (271.8)	1031.0 (273.1)	<0.01
TG concentration, mg/dl	103.3 (66.0)	81.4 (41.4)	75.2 (39.6)	75.7 (37.0)	<0.01
BMI, kg/m^2^	26.6 (5.8)	25.2 (5.2)	24.3 (4.6)	24.3 (4.5)	<0.01
Waist circumference, cm	83.4 (13.9)	79.6 (11.9)	77.6 (10.7)	77.9 (10.0)	<0.01
Systolic blood pressure, mm Hg	109.5 (11.1)	108.4 (10.3)	106.0 (10.4)	106.3 (10.1)	<0.01
Diastolic blood pressure, mm Hg	69.5 (10.3)	67.6 (8.7)	66.6 (8.7)	66.3 (8.9)	<0.01
Serum glucose, mg/dl	89.3 (22.3)	84.8 (10.5)	83.6 (9.7)	83.0 (9.4)	<0.01
PA intensity score	348.6 (273.3)	402.1 (301.5)	394.3 (273.7)	394.1 (293.8)	<0.01
HEI score (0–100)	61.1 (9.4)	62.0 (9.5)	62.9 (9.2)	62.9 (9.4)	<0.01
Saturated fats (% of energy)	14.1 (2.8)	14.2 (2.9)	14.1 (2.9)	14.1 (3.0)	0.68
Proteins (% of energy)	14.9 (2.5)	14.8 (2.6)	14.8 (2.6)	14.9 (2.8)	0.83
Carbohydrates (% of energy)	45.4 (7.3)	45.9 (7.4)	46.3 (7.1)	46.3 (7.4)	0.07
Hypertension treatment, %	35 (4.7%)	12 (1.6%)	15 (2.0%)	16 (2.2%)	<0.01
Diabetes treatment, %	10 (1.3%)	6 (0.8%)	5 (0.7%)	8 (1.1%)	0.56
Prevalent diabetes, %	21 (2.8%)	9 (1.2%)	6 (0.8%)	8 (1.1%)	<0.01
Prevalent hypertension, %	53 (7.0%)	21 (2.8%)	18 (2.4%)	20 (2.7%)	<0.01
Current smoking, %	203 (27.1%)	179 (24.0%)	171 (22.9%)	204 (27.8%)	0.09
Regular alcohol use, %	445 (71.0%)	454 (71.8%)	455 (70.8%)	469 (72.7%)	0.86
Prevalent coronary artery calcification >0 at Y25 exam, %	257 (40.0%)	173 (26.5%)	138 (21.9%)	148 (24.5%)	<0.01

Heavy drinking defined as >1 drink per day for women and >2 drinks per day for men. Continuous measures presented as mean and SD in parentheses. Categorical variables are presented as number and percentage in parentheses.

The Y20 characteristics of quartiles of annualized rate of change are presented in Table 4. The higher rate of change quartiles had a higher HDL-C and higher concentrations of atherogenic cholesterol fractions. Interestingly, the TG concentrations were highest in the lowest and highest apoB change quartiles. Those in the higher rate of change groups had lower Y20 systolic blood pressures, lower BMI and waist circumference, and lower serum glucose levels than were observed in the lower rate of change groups. There were higher HEI and PA scores in participants in the highest rate of change quartiles when compared with the lowest rate of change. Regular alcohol use was higher in the higher rate of apoB change quartiles.

**Table 4:** Y20 characteristic comparison by quartile of annualized apoB concentration change (last–first apoB/years between measurements)

Variable	0–25% (*n* = 763)	25–50% (*n* = 764)	50–75% (*n* = 764)	75–100% (*n* = 764)	*P*
Annualized apoB change, mg/dl/year	−0.7 (0.7)	0.3 (0.2)	0.8 (0.2)	1.7 (0.6)	<0.01
First apoB, mg/dl	102.7 (21.0)	84.8 (16.9)	77.9 (15.6)	76.1 (16.1)	<0.01
Last apoB, mg/dl	86.1 (17.0)	91.7 (16.9)	98.1 (15.7)	114.6 (20.8)	<0.01
Black, %	361 (47.3%)	363 (47.5%)	327 (42.8%)	320 (41.9%)	0.05
Male, %	364 (47.7%)	361 (47.3%)	310 (40.6%)	306 (40.1%)	<0.01
Age, years	45.7 (3.5)	45.1 (3.6)	45.1 (3.6)	45.1 (3.6)	<0.01
Educational status, years	14.9 (2.6)	15.0 (2.5)	15.4 (2.5)	15.1 (2.6)	<0.01
Total cholesterol, mg/dl	186.8 (35.3)	183.5 (32.4)	186.0 (31.3)	199.8 (34.8)	<0.01
HDL-C, mg/dl	51.8 (15.3)	53.7 (15.2)	55.3 (15.2)	54.3 (15.0)	<0.01
Non-HDL–C, mg/dl	134.9 (35.7)	129.8 (32.9)	130.7 (31.4)	145.6 (35.0)	<0.01
LDL–C, mg/dl	112.7 (31.5)	110.6 (29.0)	112.9 (28.7)	125.2 (31.3)	<0.01
LDL-P concentration, nmol/l	1310.5 (393.3)	1251.0 (368.4)	1249.5 (344.0)	1402.5 (387.9)	<0.01
TG concentration, mg/dl	124.1 (95.7)	105.7 (70.0)	98.1 (57.8)	113.6 (70.3)	<0.01
BMI, kg/m^2^	30.5 (7.8)	29.4 (6.8)	28.6 (6.7)	28.8 (6.3)	<0.01
Waist circumference, cm	95.5 (17.6)	91.6 (15.4)	89.5 (15.1)	90.6 (13.3)	<0.01
Systolic blood pressure, mm Hg	117.1 (15.5)	115.7 (14.5)	114.1 (14.4)	115.7 (13.8)	<0.01
Diastolic blood pressure, mm Hg	73.5 (11.9)	72.3 (10.9)	70.8 (10.7)	72.1 (10.6)	<0.01
Serum glucose, mg/dl	97.3 (33.3)	90.5 (18.3)	89.0 (15.1)	90.1 (21.2)	<0.01
PA intensity score	301.5 (252.3)	344.5 (287.4)	353.3 (269.1)	352.9 (290.2)	<0.01
HEI score (0–100)	68.9 (10.4)	69.4 (10.8)	70.8 (10.0)	70.1 (10.3)	<0.01
Saturated fats (% of energy)	11.3 (3.0)	11.7 (3.3)	11.4 (3.2)	11.8 (3.1)	0.01
Proteins (% of energy)	15.8 (4.0)	15.4 (3.7)	15.4 (3.4)	15.6 (3.6)	0.12
Carbohydrates (% of energy)	47.1 (10.1)	47.0 (9.4)	47.4 (9.2)	46.3 (9.4)	0.16
Hypertension treatment, %	197 (25.9%)	120 (15.7%)	104 (13.6%)	111 (14.5%)	<0.01
Diabetes treatment, %	103 (13.5%)	55 (7.2%)	35 (4.6%)	38 (5.0%)	<0.01
Prevalent diabetes, %	119 (15.6%)	62 (8.1%)	42 (5.5%)	42 (5.5%)	<0.01
Prevalent hypertension, %	243 (31.9%)	155 (20.3%)	134 (17.6%)	138 (18.1%)	<0.01
Current smoking, %	159 (21.0%)	140 (18.4%)	119 (15.8%)	149 (19.7%)	0.06
Regular alcohol use, %	370 (49.8%)	392 (52.3%)	420 (56.0%)	447 (59.7%)	<0.01
Prevalent coronary artery calcification >0 at Y25 exam, %	257 (40.0%)	173 (26.5%)	138 (21.9%)	148 (24.5%)	<0.01

Heavy drinking defined as >1 drink per day for women and >2 drinks per day for men. Continuous measures presented as mean and SD in parentheses. Categorical variables are presented as number and percentage in parentheses.

Spaghetti plots of intraindividual change, stratified by baseline apoB quartile and annualized rate of change quartile, are shown in supplemental Fig. S2. Of those in the lowest quartile of first measured apoB, the mean (SD) rate of change was +1.0 (0.69) mg/dl/year. Further decreases in apoB were unusual in this baseline stratum. Of those in the middle two quartiles of baseline apoB measurement, the distribution across strata of change was more balanced than was seen in the highest or lowest baseline strata. In those in the highest baseline stratum of apoB, the mean (SD) rate of change was −0.2 (1.1) mg/dl/year.

### Baseline and cumulative predictors of rate of change

The beta coefficients of linear regression models assessing the participant characteristics that are associated with rates of change in apoB are presented in Table 5. In multivariable analysis, lower first measured apoB, female sex, white race, lower BMI, and current tobacco use at the Y2 exam were significant predictors of a higher rate of change in apoB across early adulthood. When cumulative participant characteristic levels across follow-up were considered (model 4) in multivariable modeling, female sex, white race, lower HDL-C, lower glucose, and alcohol use were associated with an increasing annualized rate of apoB change.

**Table 5:** Summary results presented as beta coefficients with 95% from linear regression models for annualized apoB change (0.1 mg/dl/year) by participant characteristic levels

Participant characteristic	Model 1	Model 2	Model 3	Model 4
ApoB (per 1 mg/dl)	−0.25 (−0.26, −0.23)^a^	−0.25 (−0.26, −0.23)^a^	−0.24 (−0.25, −0.22)^a^	−0.24 (−0.26, −0.23)^a^
Age (per 1 year)		−0.003 (−0.07, 0.06)	0.02 (−0.0, 0.10)	0.02 (−0.05, 0.09)
Male *vs.* female		−0.87 (−1.48, −0.25)^a^	−0.78 (−1.57, −0.01)^a^	−1.63 (−2.41, −0.85)^a^
Black *vs.* white		−1.25 (−1.89, −0.60)^a^	−0.84 (−1.57, −0.12)^a^	−0.76 (−1.47, −0.06)^a^
Maximum education, per 1 year		−0.09 (−0.21, 0.03)	−0.10 (−0.23, 0.04)	−0.09 (−0.22, 0.04)
HDL, mg/dl			−0.01 (−0.03, 0.02)	−0.04 (−0.06, −0.01)^a^
Systolic blood pressure, mm Hg			−0.01 (−0.03, 0.03)	0.02 (−0.00, 0.06)
BMI, kg/m^2^			−0.09 (−0.15, −0.02)^a^	−0.05 (−0.11, 0.003)^b^
Glucose, mg/dl			−0.06 (−0.08, −0.04)	−0.02 (−0.05, −0.01)^a^
Diabetes treatment, yes *vs.* no			−0.09 (−2.44, 2.27)	−0.07 (−0.17, 0.03)
Hypertension treatment, yes *vs.* no			−0.21 (−2.41, 1.99)	−0.09 (−0.18, 0.004)^b^
Current smoker, yes *vs.* no			0.93 (0.158,1.70)^a^	0.15 (−0.11, 0.41)
Current drinker, yes *vs.* no			0.09 (−0.62, 0.79)	0.04 (0.002, 0.07)^a^
PA intensity score			0.29 (−0.05, 0.63)^b^	0.29 (−0.04, 0.63)^b^
HEI			0.30 (−0.0, 0.65)^b^	0.09 (−0.25, 0.44)

Model 1–3: model adjustment with baseline covariates; model 4 was adjusted with cumulative risk factors. The adjusted cumulative risk factors are as follows: HDL per year, mg/day; systolic blood pressure per year , mm Hg; BMI per year, kg/m^2^; glucose per year, mg/dl; cumulative years of diabetes treatment, hypertension treatment; PA intensity score per year; average of HEI index (0–100).

a Significant *P* < 0.05.

b Borderline significant *P* < 0.10.

#### Sensitivity analyses

The characteristics of those included versus excluded participants are presented in supplemental Table S2. When using an imputed dataset, we observed similar findings regarding to the mean and range of apoB across all exam years, and the average annualized rate of apoB change between first and last measured apoB. In multivariable models, all the predictors as well as their relative strength of association with apoB change were similar to the unimputed dataset. When CARDIA participants who initiated lipid-lowering therapy during follow-up were removed from the analysis dataset, the mean BMI, TG level, glucose, and DM prevalence in the lowest rate of change quartile at Y20 were lower than observed in the primary analysis, suggesting that some of the decrease in apoB in that group was due to the use of lipid-lowering therapy in higher risk individuals. However, removal of treated participants resulted in very minor differences in the annualized rates of change and had no effect on multivariable predictors of apoB change over time.

### Comparison of the variation in rates of change between apoB and non-HDL-C, LDL-C, and LDL-P

**Fig. 4:**
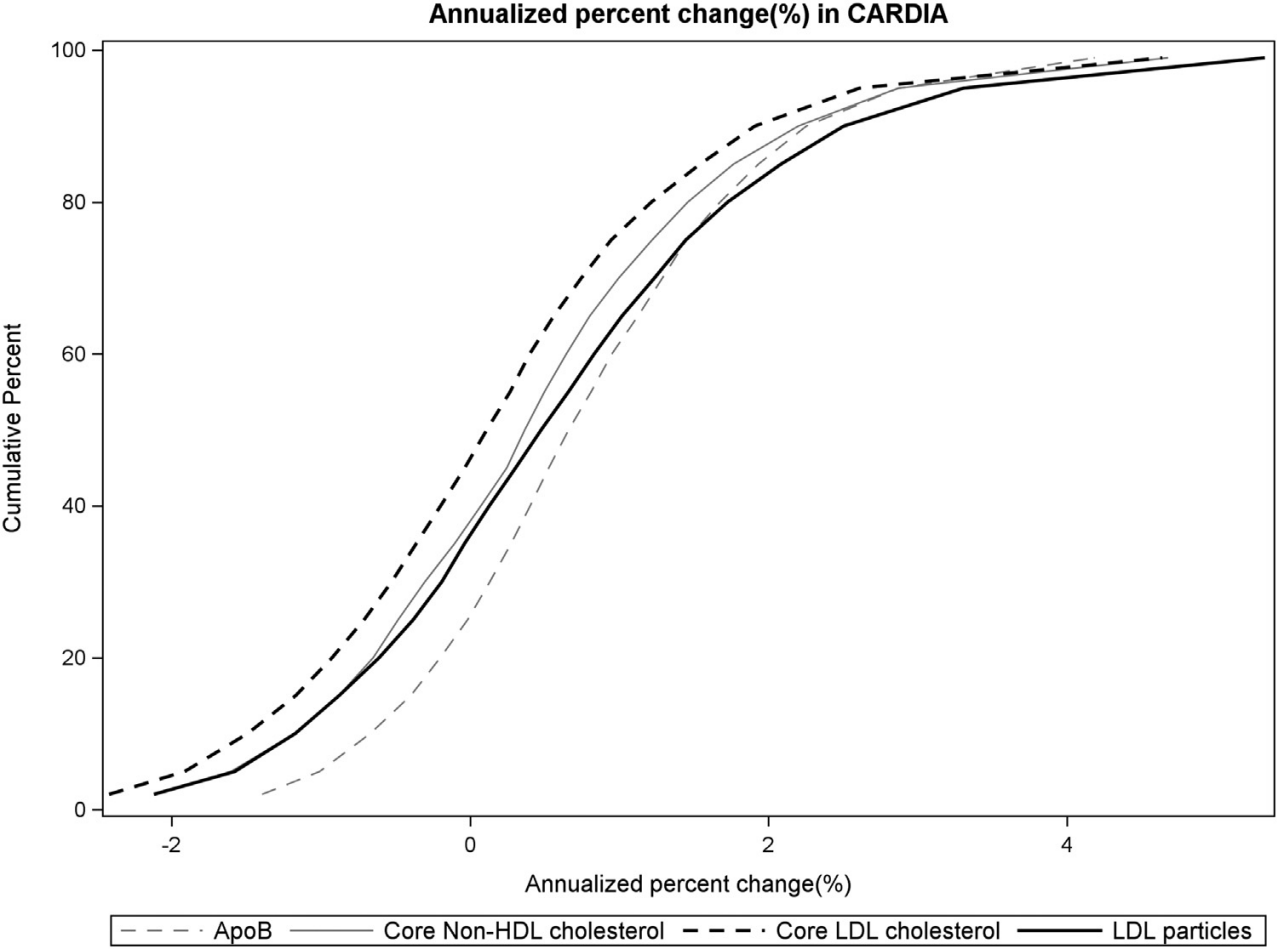
Cumulative percent distribution of the annualized percent change in apoB, non-HDL-C, LDL-C, and LDL-P number.

The distributions of percent annual change for apoB, non-HDL-C, LDL-C, and LDL-P are shown in Fig. 4. The ranges of percent change for LDL-P, LDL-C, and non-HDL-C are larger than the range of percent change for apoB. The SDs of the percent annualized rate of change for apoB, non-HDL-C, LDL-C, and LDL-P were 1.2, 1.4, 1.5, and 1.7, respectively. As shown in Fig. 4, close to 40% of CARDIA participants had a negative annualized percent change in non-HDL-C, LDL-C, and LDL-P during early adult life, whereas approximately 20% of CARDIA participants had a negative annualized percent change in apoB. The mean percent change was +0.77, +0.46, +0.20, and +0.62 for apoB, non-HDL-C, LDL-C, and LDL-P, respectively. Across all exams, the absolute ranges of non-HDL-C, LDL-C, and LDL-P values are greater than apoB, thus the modest differences in percent change in LDL-C, non-HDL-C, and LDL-P reflect, on average, larger annual changes in absolute value over time in these measures (per percent change) when compared with apoB.

## Discussion

Leveraging unique data from a well-phenotyped cohort of young adults followed for two decades with multiple measures of apoB, we describe distributions of apoB concentrations and demonstrate only modest differences in distributions by sex across young adulthood. We also observed that apoB levels are dynamic across the early adult life course, and intraindividual change in apoB over time tended to be linear. The mean (SD) rate of change was 0.52 (1.0) mg/dl/year, with a range of −6.26 to +9.21 mg/dl/year. The baseline apoB concentration was significantly and inversely associated with apoB change, whereas female sex, lower BMI, and higher HDL-C level had more modest associations with increasing apoB during the early adult life course. The substantial interindividual variation in apoB change over time as well as the relatively modest associations between baseline clinical characteristics (other than first apoB measurement) and apoB change suggest that commonly measured clinical characteristics (at least at one time point) are unlikely to predict future apoB levels well.

The cross-sectional distributions of apoB that we observed at exam years in CARDIA are consistent with distributions that were reported in the National Health and Nutrition Examination Survey (NHANES) III study (23). However, our study is the first description of untreated intraindividual change in apoB concentration across 28 years of the early adult life course. In NHANES III study, differences in the mean apoB concentration between the 20- to 30- and 40- to 50-year-old age groups were 21 mg/dl for men and 8 mg/dl for women, which suggested that apoB levels increase with age (23). However, NHANES III was a serial cross-sectional survey, and the different age ranges were not derived from a cohort. Thus, differences in apoB concentration by age group observed in NHANES III study could be due to birth cohort effects and differences in the sampling of individuals that represented different age groups. Thus, the patterns and rates of change in apoB concentration and their correlates that we report in this article could not have been determined from the NHANES III cross-sectional data alone.

ApoB blood concentrations are determined by the rates at which apoB lipoproteins are produced and cleared from the plasma (2, 24). In most individuals, the majority of apoB lipoproteins present in the blood are VLDL and LDL-Ps. Thus, the rates of production and clearance of VLDL and LDL-Ps determine most of the intraindividual and interindividual differences in apoB. Although the mediators of apoB synthetic rate and VLDL particle assembly are not completely understood, feeding and lipid kinetic studies have demonstrated that overnutrition, exogenous intake of saturated fats and simple carbohydrates, and/or insulin resistance causes increases in VLDL particles and therefore increased total apoB (25, 26, 27). Thus, chronic excess caloric intake, weight gain, and insulin resistance that occurred during follow-up likely account for some of the apoB increases observed in this analysis. Binding of the apoB molecules present on LDL-Ps to the LDL receptor causes removal of the LDL-P from the serum and downregulation of cholesterol synthesis (28). Therefore, to the extent that removal of LDL-Ps depends on the activity of the LDL receptor pathway, a decrease in LDL receptor density with age may *partially* explain the trend to higher levels of apoB seen in many individuals. However, apoB is present on remnant, VLDL, and Lp(a) as well as LDL-Ps. The relative molar concentration of each of these lipoprotein species can vary within and across individuals over time, and some of the non-LDL apoB lipoprotein species are not directly cleared by the LDL receptor (29). Therefore, change in apoB concentration should not be solely attributed to the LDL receptor density change and alternative exposures, and biologic pathways must contribute to the variation in apoB concentration observed with aging over the early adult life course.

Regression to the mean likely contributes to some of the observed patterns of change that we report, as those in the highest and lowest first apoB measurement were more likely, on average, to have repeated values that were closer to normative apoB values. Repeated measures of biological phenomena typically provide a more accurate estimate of an individual’s “usual” values; the extent to which this phenomenon is driven by measurement error versus true biologic variation cannot be determined from this study. Nonetheless, regression to the mean is seen in clinical practice when serial lipid testing is performed in individual patients. Thus, our findings are instructive and inform what may be seen in individual patient care.

The substantial changes observed over time, as well as the modest associations with baseline predictors reported above, suggest that clinicians will not be able to predict using readily available clinical information what a young adult patient’s apoB level may be 5–15 years after a one-time measurement. Thus, serial measurement would be needed to monitor a patient’s apoB exposure across early adult life. Nonetheless, the rates of change that we report from CARDIA (mean rate = 0.52 mg/dl/year) may be useful when serial apoB testing is performed, as clinicians can now have a reference value for the normative, though not necessarily optimal, rates of change in apoB concentration.

Although we observed substantial variation in apoB levels across early adult life, the intraindividual variation in apoB level across early adult life was less than that was observed for non-HDL-C, LDL-C, and LDL-P. These observations suggest that of the indices of atherogenic lipoprotein burden, apoB is the most stable during early adult life. Thus, in young adults, one-time measures of aopB may be a better (though still likely inadequate) marker of expected future cumulative atherogenic lipoprotein exposure than non-HDL-C, LDL-C, and LDL-P. However, further research is needed to determine if these differences in stability of these measures of atherogenic lipoprotein burden during early adult life translate into meaningful differences in long-term risk estimation for young adults. Similarly, the lower intrinsic variability in apoB during early adult life—in the context of its well-known mediating effect on ASCVD risk—may indicate that apoB is a better target for lifestyle optimization of lipid-lowering therapy in some.

This study has several notable strengths. First, this article represents the first description of rates of change in apoB across the early adult life course using multiple longitudinal measurements in black and white men and women in a community-based sample. Second, in addition to statistics of central tendency (mean, median, SD, etc.), we show individual-level patterns of change in the spaghetti plots and waterfall plots, which can help contextualize patient-level observations in clinical practice. Third, the quality of demographic and behavioral assessment as well as traditional risk factor measurements in the CARDIA study is excellent. Fourth, our observations of apoB are put in context of other commonly used atherogenic lipid measures.

Several limitations should be considered as well. First, CARDIA enrolled exclusively self-reported black and white Americans. Thus, it is unclear if the patterns and rates of change that we report are generalizable to other race and ethnicity groups. Second, since our interest was in describing longitudinal patterns of apoB change, we included CARDIA participants who attended the Y20 and at least two of the previous exams (Y15, Y7, or Y2). Thus, participants who were lost to follow-up or died prior to the Y20 exam were not included in this analysis, which disproportionally excluded young black men because of lower rates of follow-up in this group (30). However, sensitivity analyses that imputed missing data did not significantly change our reported results. Our inclusion criteria also excluded some participants with chronic diseases (e.g., HIV) who either died or did not attend the Y20 exam. Patterns of apoB change may be different in young individuals with severe chronic illness; thus, our results may not correctly inform the expected apoB rates of change in specific subgroups of patients with chronic illness. Third, NMR does not provide a direct measurement of apoB but a derived value from magnetic resonance decay signals of lipid methyl and methylene groups. However, we are confident in the accuracy and precision of the apoB values provided by NMR as NMR apoB measures have been previously validated against immunonephelometry on standard assays with *r*^2^ values of 0.98 (31). Furthermore, if the accuracy of the NMR apoB measure was poor but consistent, then the patterns and rates of change as well as the variance in these rates that we report would not be affected. On the other hand, if precision of apoB quantification was worse than other atherogenic lipid measures, we would have expected greater variance in apoB change than is observed in LDL-C, non-HDL-C, and LDL-P, but we observed the opposite—apoB had less variance over time than directly measured lipid values.

In summary, this article represents the first description of intraindividual apoB concentration change over the early adult life course. ApoB concentrations over time are dynamic, with the average person experiencing a +0.52 mg/dl/year increase (∼15 mg/dl over 28 years). Furthermore, the interindividual variation in change over time is substantial as well (range of −6.2 to +9.2 mg/dl/year), and the ability to predict an individual’s rate of change using one-time assessment of traditional clinical variables appears modest. However, although absolute variation of apoB blood concentration was significant across early adult life, it was less than was observed for NDHL-C, LDL-C, and LDL-P, suggesting that of these measures, apoB is the most consistent measure of atherogenic lipoprotein burden in young adults. Nonetheless, in total, these observations suggest that serial apoB testing is needed if one aims to quantify the cumulative burden of apoB atherogenic particles across early adult life. Furthermore, an improved understanding of the risks associated with different apoB concentrations, as well as the presence of potentially critical thresholds of exposure or potentially critical periods of exposure during early adult life, is needed to inform testing guidelines for this central determinant of ASCVD risk.

## Data Availability

The data that support the findings of this study are available in the CARDIA study by request.

## Supplemental Data

This article contains supplemental data.

## Conflict of Interest

Dr Wilkins reports consulting work for 3M.
