# Supplementary material for: Analysis of apoB Concentrations Across Early Adulthood and Predictors for Rates of Change Using CARDIA Study Data
Source: J Lipid Res. 2022 Oct 19;63(12):100299. doi: 10.1016/j.jlr.2022.100299 (PMC9694068; doi:10.1016/j.jlr.2022.100299)
Supplement: Supplemental Tables [file mmc1.docx]

| Supplemental Table1A: **Y7** Characteristics of CARDIA participants by apoB Concentration Quartile at the **Year 20** Examination. | | | | | |
| --- | --- | --- | --- | --- | --- |
|  | Apolipoprotein B Concentration Quartile (N)  (Range) | | | |  |
| Variable | 0-25% (n=763)  (33.1-81.7mg/dl) | 25-50% (n=764)  (81.7-93.9mg/dl) | 50-75% (n=764)  (93.9-108.0mg/dl) | 75-100% (n=764)  (108.0-196.8mg/dl) | P value |
| Black, % | 354 (46.4%) | 354 (46.3%) | 341 (44.6%) | 322 (42.1%) | 0.30 |
| Male, % | 298 (39.1%) | 297 (38.9%) | 339 (44.4%) | 407 (53.3%) | <.01 |
| Age, yrs | 32.0 (3.6) | 32.1 (3.6) | 32.3 (3.5) | 32.4 (3.5) | 0.12 |
| edu, yrs | 14.9 (2.5) | 14.8 (2.4) | 14.9 (2.5) | 14.6 (2.4) | 0.04 |
| ApoB, mg/dL* | 71.9 (18.9) | 81.7 (18.1) | 87.9 (17.2) | 102.0 (19.8) | <.01 |
| Total Cholesterol, mg/dL | 165.3 (31.6) | 180.7 (29.6) | 189.8 (28.2) | 211.4 (33.1) | <.01 |
| HDL Cholesterol, mg/dL | 56.4 (14.2) | 55.7 (13.5) | 53.7 (13.5) | 50.8 (13.3) | <.01 |
| Non-HDL Cholesterol, mg/dL | 108.9 (31.8) | 124.9 (30.0) | 136.2 (28.4) | 160.6 (33.4) | <.01 |
| LDL Cholesterol, mg/dL | 92.8 (28.2) | 109.1 (27.0) | 118.8 (25.1) | 140.3 (30.2) | <.01 |
| LDL particles concentration, nmol/L | 1073.1 (360.0) | 1255.7 (344.5) | 1374.2 (334.1) | 1627.9 (378.5) | <.01 |
| Triglyceride concentration, mg/dL | 85.8 (55.1) | 86.2 (58.1) | 95.6 (60.8) | 113.7 (74.7) | <.01 |
| Body Mass Index, kg/m2 | 25.7 (5.9) | 26.5 (6.1) | 26.8 (5.6) | 27.1 (5.6) | <.01 |
| Waist Circumference, cm | 81.1 (14.2) | 82.6 (13.9) | 83.9 (13.0) | 86.0 (13.3) | <.01 |
| Systolic Blood Pressure, mmHg | 107.0 (11.9) | 107.2 (11.5) | 108.3 (12.3) | 109.6 (11.7) | <.01 |
| Diastolic Blood Pressure , mmHg | 67.9 (10.1) | 68.6 (9.9) | 69.3 (10.5) | 69.8 (9.5) | <.01 |
| Serum Glucose, mg/dL | 89.0 (19.5) | 88.6 (14.4) | 88.4 (10.8) | 89.8 (12.7) | 0.28 |
| Physical Activity Intensity Score | 355.6 (268.9) | 336.7 (274.9) | 333.6 (269.4) | 346.1 (283.7) | 0.42 |
| Healthy Eating Index score (0-100) | 65.6 (10.1) | 65.4 (10.2) | 66.4 (9.8) | 64.1 (9.5) | < .01 |
| Saturated Fats (% of energy) | 12.0 (3.1) | 12.2 (3.1) | 12.0 (2.9) | 12.3 (3.1) | 0.13 |
| Protein (% of energy) | 14.6 (2.8) | 14.7 (2.7) | 14.7 (2.5) | 14.9 (2.7) | 0.16 |
| Carbohydrates (% of energy) | 49.8 (8.5) | 49.9 (8.6) | 49.4 (7.6) | 48.9 (7.9) | 0.06 |
| Hypertension Treatment, % | 11 (1.5%) | 14 (1.9%) | 13 (1.8%) | 8 (1.1%) | 0.59 |
| Diabetes Treatment, % | 22 (3.0%) | 33 (4.6%) | 14 (1.9%) | 24 (3.3%) | 0.04 |
| Prevalent Diabetes, % | 28 (3.8%) | 33 (4.5%) | 15 (2.1%) | 26 (3.5%) | 0.06 |
| Prevalent Hypertension, % | 28 (3.8%) | 33 (4.5%) | 44 (6.0%) | 31 (4.2%) | 0.21 |
| Current smoking, % | 172 (23.5%) | 150 (20.7%) | 177 (24.2%) | 195 (26.6%) | 0.07 |
| Regular Alcohol Use, % | 413 (56.5%) | 383 (53.0%) | 403 (55.3%) | 407 (55.8%) | 0.56 |
| HTN = hypertension; DM = diabetes mellitus; PA = physical activity; Continuous measures presented as mean and standard deviation in parentheses. Categorical variables are presented as number and percentage in parentheses. *Mean apoB concentration derived from the 2582 participant samples that were analyzed via NMR. | | | | | |

| Supplemental Table1B: **Y15** Characteristics of CARDIA participants by The Year 20 ApoB Concentration Quartile. | | | | | |
| --- | --- | --- | --- | --- | --- |
|  | Apolipoprotein B Concentration Quartile (N) (Range) | | | |  |
| Variable | 0-25% (n=763)  (33.1-81.7mg/dl) | 25-50% (n=764)  (81.7-93.9mg/dl) | 50-75% (n=764)  (93.9-108.0mg/dl) | 75-100% (n=764)  (108.0-196.8mg/dl) | P Value |
| Black, % | 354 (46.4%) | 354 (46.3%) | 341 (44.6%) | 322 (42.1%) | 0.29 |
| Male, % | 298 (39.1%) | 297 (38.9%) | 339 (44.4%) | 407 (53.3%) | <.01 |
| Age, yrs | 40.1 (3.6) | 40.3 (3.6) | 40.4 (3.6) | 40.5 (3.5) | 0.12 |
| Education Status, yrs | 15.2 (2.6) | 15.1 (2.5) | 15.1 (2.5) | 14.8 (2.5) | 0.03 |
| ApoB, mg/dL* | 77.8 (16.6) | 87.6 (15.4) | 95.8 (16.1) | 109.4 (17.8) | <.01 |
| Total Cholesterol, mg/dL | 158.1 (28.1) | 174.7 (26.7) | 186.9 (26.8) | 206.7 (30.7) | <.01 |
| HDL Cholesterol, mg/dL | 51.1 (13.1) | 51.3 (13.2) | 49.3 (13.1) | 46.3 (12.8) | <.01 |
| Non-HDL Cholesterol, mg/dL | 107.0 (27.4) | 123.3 (25.3) | 137.6 (26.2) | 160.4 (29.3) | <.01 |
| LDL Cholesterol, mg/dL | 90.5 (24.7) | 106.1 (23.4) | 118.6 (23.8) | 136.0 (27.4) | <.01 |
| LDL particles concentration, nmol/L | 962.1 (296.6) | 1130.9 (280.1) | 1273.4 (288.9) | 1519.4 (320.7) | <.01 |
| Triglyceride concentration, mg/dL | 88.5 (62.1) | 94.8 (70.2) | 105.2 (63.4) | 136.5 (90.8) | <.01 |
| Body Mass Index, kg/m2 | 27.6 (7.1) | 28.5 (7.0) | 28.9 (6.2) | 29.5 (6.3) | <.01 |
| Waist Circumference, cm | 85.9 (15.9) | 88.3 (16.0) | 89.6 (13.8) | 92.6 (13.8) | <.01 |
| Systolic Blood Pressure, mmHg | 111.3 (14.8) | 112.0 (14.2) | 113.0 (15.3) | 115.3 (14.1) | <.01 |
| Diastolic Blood Pressure, mmHg | 73.2 (11.8) | 73.6 (10.9) | 74.9 (11.4) | 75.9 (11.3) | <.01 |
| Serum Glucose, mg/dL | 91.2 (22.4) | 91.6 (20.5) | 90.4 (14.3) | 93.0 (17.8) | 0.08 |
| Physical Activity Intensity Score | 362.0 (285.4) | 336.9 (276.9) | 337.9 (273.3) | 352.6 (278.8) | 0.25 |
| Hypertension Treatment, % | 44 (6.1%) | 62 (8.6%) | 49 (6.7%) | 54 (7.5%) | 0.30 |
| Diabetes Treatment, % | 44 (6.1%) | 37 (5.1%) | 35 (4.8%) | 39 (5.4%) | 0.72 |
| Prevalent Diabetes, % | 52 (7.2%) | 45 (6.2%) | 43 (5.9%) | 51 (7.1%) | 0.69 |
| Prevalent Hypertension, % | 93 (13.0%) | 106 (14.7%) | 111 (15.2%) | 139 (19.3%) | < .01 |
| Current smoking, % | 143 (19.9%) | 113 (15.7%) | 153 (21.0%) | 167 (23.2%) | < .01 |
| Regular Alcohol Use, % | 393 (54.7%) | 378 (52.6%) | 395 (54.3%) | 365 (50.8%) | 0.45 |
| HTN = hypertension; DM = diabetes mellitus; PA = physical activity; Continuous measures presented as mean and standard deviation in parentheses. Categorical variables are presented as number and percentage in parentheses. *Mean apoB concentration derived from the 2474 participant samples that were analyzed via NMR. | | | | | |

| Supplemental Table1C: **Y30** Characteristics of CARDIA participants by The Year 20 ApoB Concentration Quartile. | | | | | |
| --- | --- | --- | --- | --- | --- |
|  | Apolipoprotein B Concentration Quartile (N)  (Range) | | | |  |
| Variable | 0-25% (n=763)  (33.1-81.7mg/dl) | 25-50% (n=764)  (81.7-93.9mg/dl) | 50-75% (n=764)  (93.9-108.0mg/dl) | 75-100% (n=764)  (108.0-196.8mg/dl) | P Value |
| Black, % | 354 (46.4%) | 354 (46.3%) | 341 (44.6%) | 322 (42.1%) | 0.30 |
| Male, % | 298 (39.1%) | 297 (38.9%) | 339 (44.4%) | 407 (53.3%) | <.01 |
| Age, yrs | 55.0 (3.6) | 55.1 (3.6) | 55.4 (3.5) | 55.4 (3.5) | 0.14 |
| Education Status, yrs | 15.5 (2.7) | 15.3 (2.6) | 15.4 (2.5) | 15.1 (2.6) | 0.04 |
| ApoB, mg/dL* | 84.8 (16.2) | 94.7 (16.9) | 102.2 (17.5) | 110.1 (21.1) | <.01 |
| Total Cholesterol, mg/dL | 176.7 (32.1) | 191.2 (34.6) | 199.7 (33.8) | 208.8 (39.0) | <.01 |
| HDL Cholesterol, mg/dL | 61.0 (17.4) | 60.1 (16.8) | 56.7 (16.6) | 52.7 (14.8) | <.01 |
| Non-HDL Cholesterol, mg/dL | 115.7 (27.9) | 131.1 (29.5) | 143.0 (30.6) | 156.1 (36.4) | <.01 |
| LDL Cholesterol, mg/dL | 98.6 (25.4) | 113.8 (28.7) | 122.6 (28.1) | 132.4 (35.0) | <.01 |
| LDL particles concentration, nmol/L | 1092.6 (289.2) | 1265.7 (295.6) | 1403.5 (315.5) | 1556.8 (382.8) | <.01 |
| Triglyceride concentration, mg/dL | 93.9 (55.2) | 96.6 (54.9) | 114.1 (69.9) | 133.8 (76.2) | <.01 |
| Body Mass Index, kg/m2 | 28.7 (6.9) | 30.1 (7.3) | 30.9 (6.8) | 31.3 (6.9) | <.01 |
| Waist Circumference, cm | 91.0 (16.6) | 94.6 (16.5) | 97.4 (15.3) | 99.8 (15.3) | <.01 |
| Systolic Blood Pressure, mmHg | 119.0 (17.6) | 120.3 (16.7) | 119.8 (15.7) | 122.6 (15.7) | <.01 |
| Diastolic Blood Pressure, mmHg | 72.7 (11.7) | 73.6 (11.1) | 73.5 (10.2) | 75.2 (10.2) | <.01 |
| Serum Glucose, mg/dL | 96.7 (30.0) | 95.1 (23.2) | 99.3 (33.5) | 102.5 (31.3) | <.01 |
| Physical Activity Intensity Score | 329.9 (266.1) | 327.3 (275.6) | 315.7 (262.2) | 323.9 (286.8) | 0.80 |
| Hypertension Treatment, % | 182 (28.4%) | 217 (33.0%) | 223 (34.2%) | 229 (35.8%) | 0.03 |
| Diabetes Treatment, % | 88 (13.8%) | 78 (11.9%) | 99 (15.2%) | 108 (16.9%) | 0.06 |
| Prevalent Diabetes, % | 89 (13.9%) | 80 (12.2%) | 105 (16.1%) | 115 (18.0%) | 0.02 |
| Prevalent Hypertension, % | 226 (35.3%) | 260 (39.5%) | 268 (41.0%) | 280 (43.8%) | 0.02 |
| Current smoking, % | 79 (12.5%) | 63 (9.7%) | 84 (13.0%) | 81 (12.8%) | 0.21 |
| Regular Alcohol Use, % | 369 (58.9%) | 351 (54.6%) | 363 (56.9%) | 357 (57.1%) | 0.48 |
| HTN = hypertension; DM = diabetes mellitus; PA = physical activity; Continuous measures presented as mean and standard deviation in parentheses. Categorical variables are presented as number and percentage in parentheses. *Mean apoB concentration derived from the 2474 participant samples that were analyzed via NMR. | | | | | |

**Supplemental Table 2. Y2 characteristics of included participants versus those excluded**

| Variable | Included (n=3055) | Excluded (n=2060) |
| --- | --- | --- |
| Black, % | 1371 (44.9%) | 1266 (61.5%) |
| Male, % | 1341 (43.9%) | 986 (47.9%) |
| Age, yrs | 27.2 (3.6) | 26.5 (3.7) |
| Education Status, yrs | 14.4 (2.3) | 13.7 (3.8) |
| Body Mass Index, kg/m2 | 25.1 (5.1) | 25.4 (5.8) |
| Waist circumference, cm | 79.6 (11.9) | 80.4 (12.8) |
| Systolic Blood Pressure, mmHg | 107.6 (10.6) | 108.4 (11.2) |
| Diastolic Blood Pressure, mmHg | 67.5 (9.3) | 67.3 (10.4) |
| Physical Activity Intensity Score | 384.7 (286.4) | 377.1 (293.2) |
| Healthy Eating Index Score (0-100) | 62.2 (9.4) | 60.6 (9.3) |
| Saturated Fats (% of energy) | 14.1 (2.9) | 14.2 (3.1) |
| Proteins (% of energy) | 14.9 (2.6) | 14.6 (2.8) |
| Carbohydrates (% of energy) | 46.0 (7.3) | 46.1 (7.7) |
| Hypertension Treatment, % | 78 (2.6%) | 45 (2.8%) |
| Diabetes Treatment, % | 29 (1.0%) | 26 (1.6%) |
| Prevalent Diabetes, % | 44 (1.5%) | 26 (1.6%) |
| Prevalent Hypertension, % | 112 (3.7%) | 70 (4.3%) |
| Current smoking, % | 757 (25.4%) | 601 (37.1%) |
| Regular Alcohol Use, % | 1823 (71.6%) | 936 (70.7%) |
