# Supplementary material for: Analysis of apoB Concentrations Across Early Adulthood and Predictors for Rates of Change Using CARDIA Study Data
Source: J Lipid Res. 2022 Oct 19;63(12):100299. doi: 10.1016/j.jlr.2022.100299 (PMC9694068; doi:10.1016/j.jlr.2022.100299)

Supplemental Figure 1: Cumulative percent distribution plot of annualized apoB change

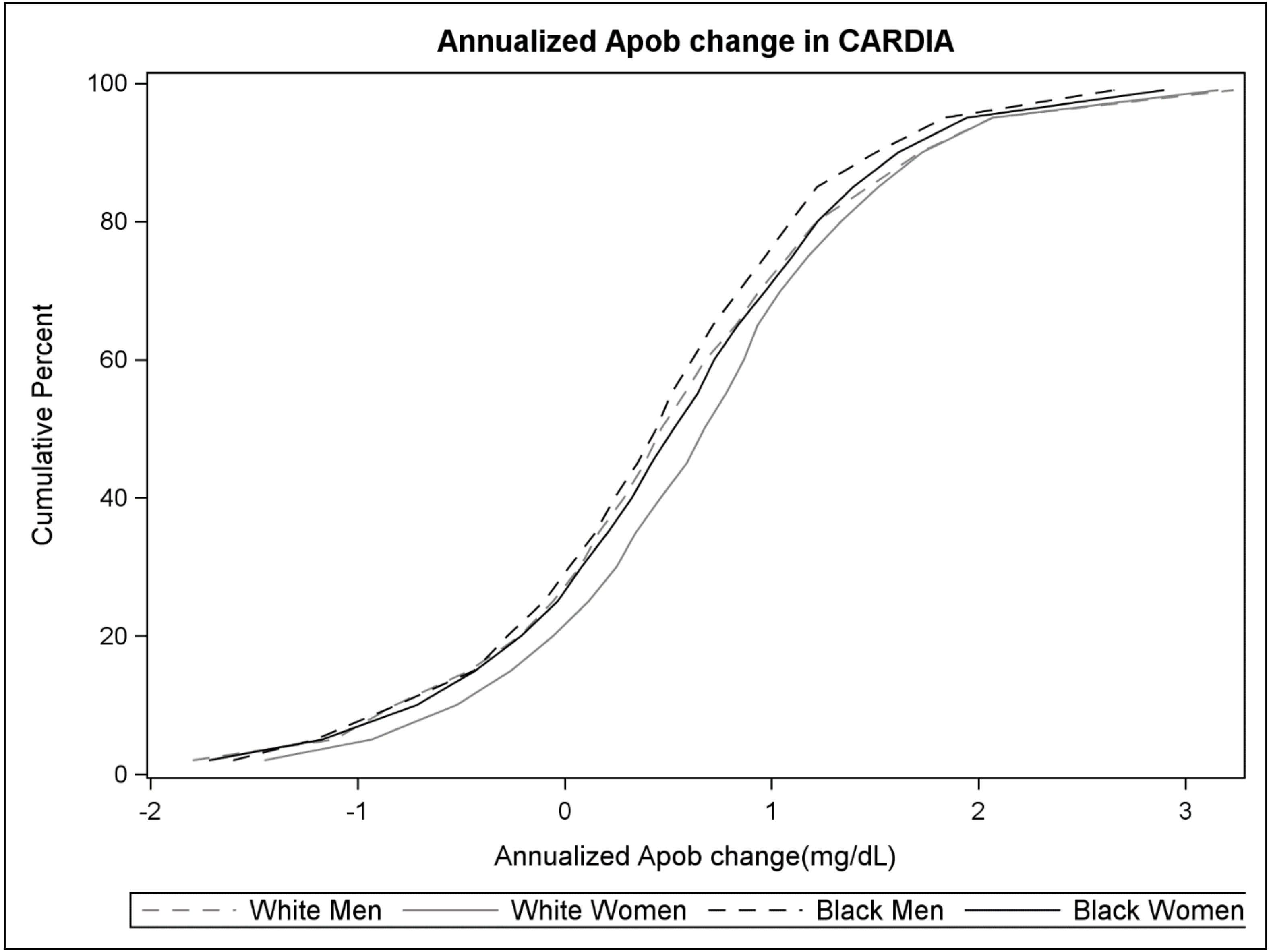

Supplemental Figure 2: Individual-Level Rates of ApoB Change Stratified by baseline level and annualized rate of change

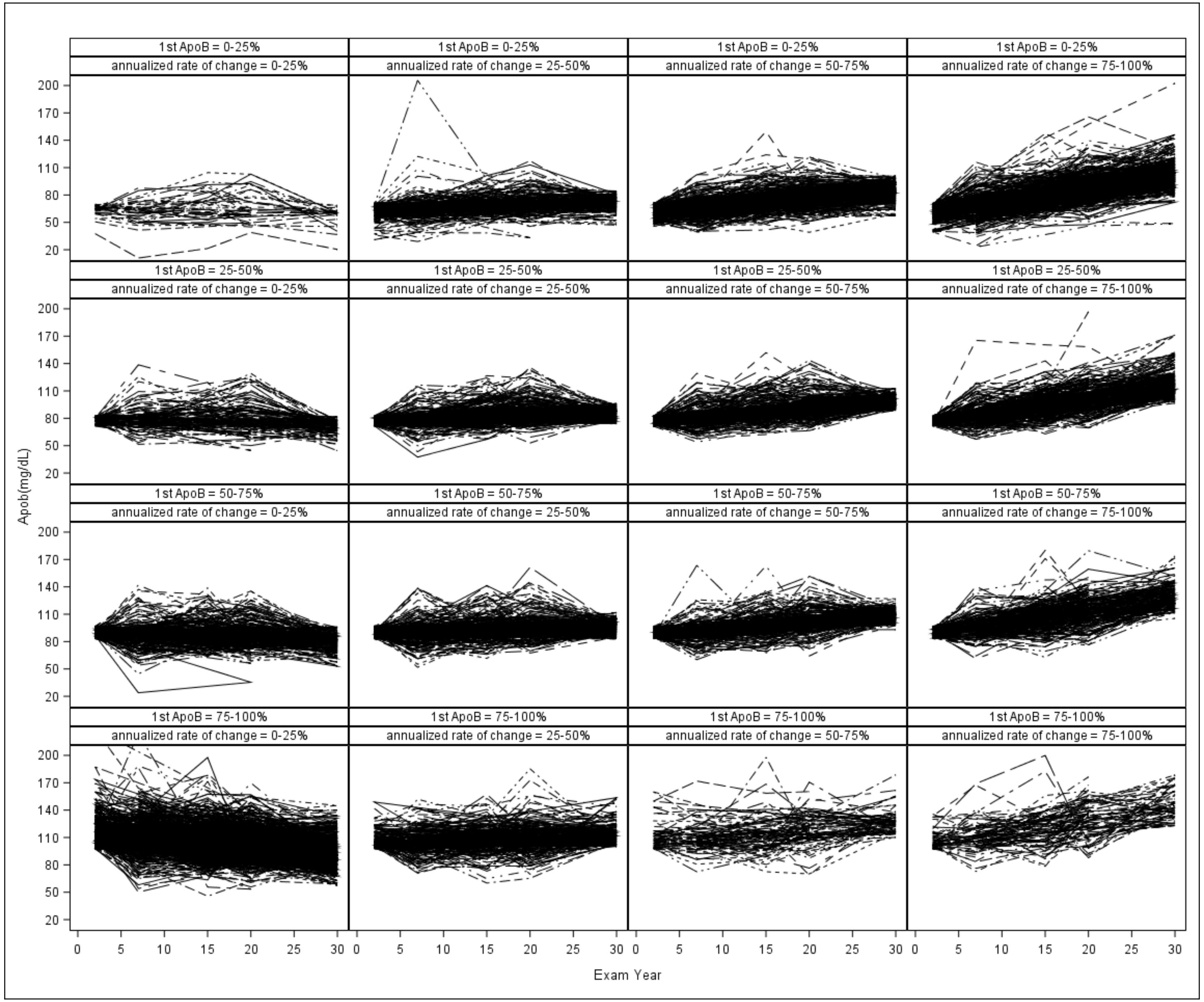

Supplement: Supplemental figures [file mmc2.pdf]
